# Improvement of Hybrid Electrode Material Synthesis for Energy Accumulators Based on Carbon Nanotubes and Porous Structures

**DOI:** 10.3390/mi14071288

**Published:** 2023-06-23

**Authors:** Boris V. Malozyomov, Vladislav V. Kukartsev, Nikita V. Martyushev, Viktor V. Kondratiev, Roman V. Klyuev, Antonina I. Karlina

**Affiliations:** 1 Department of Electrotechnical Complexes, Novosibirsk State Technical University, 630073 Novosibirsk, Russia; 2 Department of Informatics, Institute of Space and Information Technologies, Siberian Federal University, 660041 Krasnoyarsk, Russia; 3 Department of Information Economic Systems, Institute of Engineering and Economics, Reshetnev Siberian State University of Science and Technology, 660037 Krasnoyarsk, Russia; 4 Digital Material Science: New Materials and Technologies, Bauman Moscow State Technical University, 105005 Moscow, Russia; 5 Scientific Department, Kh. Ibragimov Complex Institute of the Russian Academy of Sciences, 364906 Grozny, Russia; 6 Laboratory of Geochemistry of Ore Formation and Geochemical Methods of Prospecting, A. P. Vinogradov Institute of Geochemistry of the Siberian Branch of the Russian Academy of Sciences, 664033 Irkutsk, Russia; 7 Technique and Technology of Mining and Oil and Gas Production Department, Moscow Polytechnic University, 107023 Moscow, Russia; 8 Stroytest Research and Testing Center, Moscow State University of Civil Engineering, 129337 Moscow, Russia

**Keywords:** catalyst, hybrid materials, nanotubes, electrodes, lithium-ion battery

## Abstract

Carbon materials are promising for use as electrodes for supercapacitors and lithium–ion batteries due to a number of properties, such as non-toxicity, high specific surface area, good electronic conductivity, chemical inertness, and a wide operating temperature range. Carbon-based electrodes, with their characteristic high specific power and good cyclic stability, can be used for a new generation of consumer electronics, biomedical devices and hybrid electric vehicles. However, most carbon materials, due to their low electrical conductivity and insufficient diffusion of electrolyte ions in complex micropores, have energy density limitations in these devices due to insufficient number of pores for electrolyte diffusion. This work focuses on the optimization of a hybrid material based on porous carbon and carbon nanotubes by mechanical mixing. The purpose of this work is to gain new knowledge about the effect of hybrid material composition on its specific capacitance. The material for the study is taken on the basis of porous carbon and carbon nanotubes. Electrodes made of this hybrid material were taken as an object of research. Porous carbon or nitrogen-containing porous carbon (combined with single-, double-, or multi-layer carbon nanotubes (single-layer carbon nanotubes, bilayer carbon nanotubes or multilayer carbon nanotubes) were used to create the hybrid material. The effect of catalytic chemical vapor deposition synthesis parameters, such as flow rate and methane-to-hydrogen ratio, as well as the type of catalytic system on the multilayer carbon nanotubes structure was investigated. Two types of catalysts based on Mo_12_O_28_ (μ_2_-OH)_12_{Co(H_2_O)_3_}_4_ were prepared for the synthesis of multilayer carbon nanotubes by precipitation and combustion. The resulting carbon materials were tested as electrodes for supercapacitors and lithium ion intercalation. Electrodes based on nitrogen-containing porous carbon/carbon nanotubes 95:5% were found to be the most efficient compared to nitrogen-doped porous carbon by 10%. Carbon nanotubes, bilayer carbon nanotubes and multilayer carbon nanotubes synthesized using the catalyst obtained by deposition were selected as additives for the hybrid material. The hybrid materials were obtained by mechanical mixing and dispersion in an aqueous solution followed by lyophilization to remove water. When optimizing the ratio of the hybrid material components, the most effective porous carbon:carbon nanotubes component ratio was determined.

## 1. Introduction

Carbon materials are extremely promising at present. The main areas in which they can be used are electrodes for supercapacitors and applications in lithium-ion batteries [1,2].

The battery consists of a negative electrode and a positive electrode, usually lithium, separated by a lithium-ion conducting electrolyte, such as a solution of LiPF_6_ in ethylene carbonate diethyl carbonate [2,3].

The development of new nanoelectrodes for lithium-ion intercalation contributes to improved battery life through new reactions occurring within the material volume, a more developed electrode/electrolyte contact area that results in higher charge/discharge rates; short path lengths for electron transport (allowing low electron conductivity or higher power operation); and short path lengths for Li^+^ transport (which allow operation with low conductivity materials) [3].

In addition, existing battery electrodes have a number of disadvantages compared to supercapacitors, such as undesirable electrode/electrolyte reactions due to large surface area, leading to self-discharge, poor cycling leading to a limited lifetime, and poor particle packing, contributing to lower bulk energy densities. Given these advantages and disadvantages, the development of new electrode materials for batteries is an urgent task at present [3].

**Figure 1:**
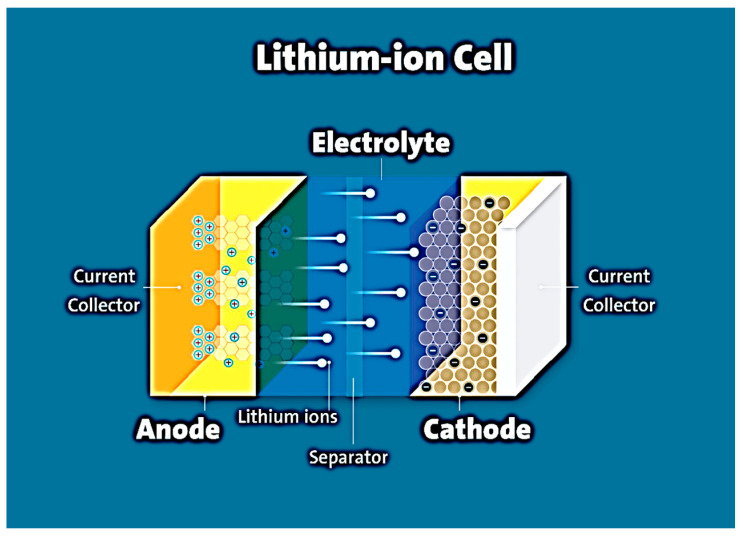
Electron and ion generation occurring on the lithium surface.

A battery consists of three components: electrodes, electrolyte, and current collectors. The positively charged electrode (cathode) provides lithium cations inside the cell (charging process), the electrons being transported through an external electric circuit. The negatively charged electrode (anode) generates lithium ions (Li^+^) into the electrolyte, which are absorbed by the cathode during the discharge process [4]. Lithium, lithium iron, or cobalt oxides are usually used as the cathode, and carbon materials (graphite, carbon nanotubes (CNT), hybrid carbon materials, etc.) are used as the anode [3,4] The cathode material and the anode material, applied to copper foil or nickel foam, are separated by an electrolyte-soaked separator. The electrolyte most commonly used is a liquid solution containing a lithium salt (LiPF_6_, LiClO_4_) dissolved in an organic solvent or mixture of organic compounds (dimethyl carbonate, ethylene carbonate, etc.), which allows lithium ions to diffuse from one electrode to another [5]. The electrolyte must be stable in the presence of both electrodes. Current collectors are usually made of metal (copper, aluminum, etc.). During battery charging, lithium ions intercalate into the carbon material. As a result, the cathode material is oxidized and the anode material is reduced. During the discharge of the lithium-ion cell, the anode is electrochemically oxidized, thereby releasing lithium ions. At the same time, electrons pass through the external circuit to the cathode. When the battery is recharged, the reverse process occurs. The characteristics of the battery depend on the chemical composition and structure of the anode and cathode and therefore on the parameters of the electrochemical processes in the battery. A diagram of a lithium-ion battery is shown in Figure 1.

Li ↔ Li^+^ + e^−^ (1)

The reversibility of lithium ion intercalation/deintercalation processes in the cell was thought to promote multiple use of batteries through the charge/discharge cycle, but in the actual process, various losses in the interaction of lithium with electrode materials and electrolytes inevitably occur. During the first charge/discharge phase, an SEI (solid-electrolyte interphase) is formed as a result of the reaction between the electrode material and the electrolyte, which allows free diffusion of lithium, preventing the intercalation of the solvent into the electrode. In the process of SEI formation, some amount of lithium is lost, which causes the appearance of irreversible battery capacity [4].

In the second step, lithium intercalation/deintercalation occurs in the carbon material through the chemical reduction of carbon, which is described by the following reaction (2):

Li^+^ + e^−^ +xC ↔ LiCx (2)

Similarly, the morphology, microstructure, and crystallinity of carbon affect the quality of the intercalated compound. Different carbon materials have different interlayer spacing of carbon layers and thickness of stacked layers, which results in different lithiation abilities [6].

Another important parameter is Coulomb efficiency, that is, the ratio of the lithium deintercalation capacity to the lithium intercalation capacity in the same cycle.

Electrolyte decomposition and physical or chemical changes in the active electrode materials can reduce the Coulomb efficiency value. The capacity of the battery varies with different current draws. At low amperage feed values, intercalation into the anode material may be less effective, thereby reducing the capacity of the material. At high amperage feed values, the anode material may degrade [2,3,4].

The pore size of the electrode materials that showed the maximum specific electrochemical capacitance was very close to the size of the electrolyte ion (relative to the electrolyte with ionic liquid). However, an increase in pore size can also change the average distance between the pore wall and the center of the ion, which contributes to a decrease in the capacitance of materials with larger pore sizes in accordance with Equation (3):

(3) $$C=\frac{A\varepsilon }{4\pi d},$$

where A is the active area of the electrode, ε is the dielectric permittivity, d is the effective thickness of the electrical double layer.

The pore distribution, pore size, and total specific surface area (m^2^/g) of the material affect the specific electrochemical capacitance. Consequently, the capacitance of the pseudocapacitor strongly depends on the electrode surface area available to the electrolyte. In Table 1, we can see the main characteristics of batteries [7,8].

Electrodes based on hybrid carbon materials exhibit high electrical conductivity, high specific electrochemical capacitance, enhanced charge-discharge capability, and improved cyclic stability. Hybrid carbon materials are electrochemically active. They can be used for high-efficiency lithium-ion batteries, supercapacitors, fuel cells, and other types of energy storage and conversion devices [3,4]. The important properties of these materials, which determine their scope of application, are chemical inertness, large specific surface area, low toxicity, and a wide range of operating temperatures [9]. Such accumulators and supercapacitors can be used in various industrial and technical devices [4]. The most promising area of battery and supercapacitor application is hybrid electric vehicles.

However, along with the positives, there are disadvantages to these materials. Most carbon materials used for supercapacitors and lithium-ion batteries have energy density limitations [10,11]. This is caused by low electrical conductivity and insufficient diffusion of electrolyte ions in complex micropores [4]. Increasing the electrical conductivity and diffusion of electrolyte ions in complex micropores is an extremely urgent task to improve the performance properties of these materials.

Hybrid materials are an attempt to combine the best properties of two components by analogy with composite materials [12]. A hybrid material attempts to retain the necessary best properties of two or more individual components, but their drawbacks are eliminated or reduced. There are quite a few works in this direction. Thus, the authors of [13] obtained a hybrid material based on transition metal oxides and single-layer carbon nanotubes-SWCNTs (MnO_2_/SWCNTs). The resulting material does not contain a binder. It was obtained by ultrasonic treatment of transition metal nanowire oxides in an isopropyl alcohol solution. Dispersion of this material was performed on a membrane of SWCNT/Al film O_23_. The initial single-layer carbon nanotubes had a specific capacitance of 80 F/g. The hybrid material based on them already has a specific capacitance of 253 F/g [13]. A good contact of SWCNTs with oxides provided this growth of the property. Ultrasonic treatment produced an optimal distribution of nanowires. All this created optimal conditions for charge transfer.

The methods for obtaining hybrid materials can be different. The sol-gel method can also be used for their production. The authors of [14] obtained a hybrid material based on titanium oxide TiO_2_ and carbon nanotubes (CNT) by sol-gel method. The specific capacitance was increased to 145 F/g at a sweep rate of 5 mV/c. In Li-ion intercalation, such material has a reversible capacity of up to 200 mAh/g [14]. Another method used to create hybrid materials is chemical vapor deposition (CVD). The material in [14] based on micro-layered carbon and CNT was obtained via this method. Its application as electrodes for supercapacitors [2] produced good results. The resulting material had a specific electrochemical capacity of 210 F/g at a current density of 3 A/g. Similar CVD treatment of phosphoric–tungstic acid and activated carbon also produced good results. Supercapacitor electrodes made of this hybrid material (phosphoric–tungstic acid and activated carbon) showed an increase in electrochemical capacity up to 254 F/g. At the same time, the electrochemical capacity of activated carbon is (185 F/g) at 2 mV/s [14].

Dispersion in an aqueous solution with ultrasonic (US) treatment made it possible to obtain [9] hybrid material. The authors took graphene oxide and multilayer carbon nanotubes (MWNTs) as the basis. Dispersion in an aqueous solution with ultrasonic (US) treatment in an ice bath produced a colloidal solution. The solution was then vacuum-filtered through a membrane filter. The mass ratios of the components of the hybrid material based on graphene oxide and MWNT were 2:1 and 1:1, respectively [9]. The initial graphene oxide and hybrid material were investigated in Li-ion intercalation. The graphene oxide/MUNT composition (2:1) showed the best reversible specific capacity at the first cycle of 439 mAh/g and a high efficiency of 98.5% (429 mAh/g specific capacity after 100 cycles) compared to the graphene oxide/MUNT composition (1:1) (548 mAh/g in the first cycle) with a specific capacity of 401 mAh/g after 100 cycles (97.6% efficiency) and with the original graphene oxide (431.9 mAh/g at the first cycle, 90.1% efficiency at 100 cycles) at 372 mA/g current density [9].

One of the most effective ways to improve the electrochemical performance of lithium-ion batteries and supercapacitors is to develop effective new hybrid materials. Mechanical mixing of initial components makes it possible to obtain such materials at minimal cost. In addition, in the process of mechanical mixing, it is possible to control the composition of the material. Such control makes it possible to set the required material characteristics.

The material, which has a predominantly microporous structure, has a developed surface area. The size of micropores is comparable to the size of electrolyte molecules, which plays a key role in selective adsorption processes, thus limiting diffusion and creating a molecular sieve effect, which is a key factor for the synthesis of new electrode materials.

The purpose of this work is to gain new knowledge about the effect of the hybrid material composition on its specific capacitance material for the study is taken on the basis of porous carbon and carbon nanotubes. Electrodes made of this hybrid material are taken as an object of research.

On the basis of the goal, the following tasks were completed in this study:

1. Synthesis of multilayer carbon nanotubes using cobalt polymolybdate. These nanotubes serve as a source of catalytic particles in the experimental work. Moreover, this is the novelty of the work.
2. Obtaining a hybrid material using the developed methodology. The material is made on the basis of carbon nanotubes and porous carbon.
3. Study of the electrochemical properties of the manufactured hybrid material.

The research significance of this work is devoted to catalytic chemical vapor deposition (CCVD) synthesis, i.e., catalytic chemical vapor deposition of carbon nanotubes from the gas phase, namely:

1. Investigate the effect of CCVD-synthesis parameters such as flow rates, methane-to-hydrogen ratios, and type of catalytic system on the structure of multi-layer carbon nanotubes (MWNTs).
2. Synthesize MWNTs and prepare two types of Mo_12_O_28_ (μ_2_-OH)_12_{Co(H_2_O)_3_}_4_-based catalyst on MgO carriers via deposition (catalyst 1) and combustion (catalyst 2).
3. Develop a technique for obtaining a hybrid carbon material based on porous carbon (H1U) or nitrogen-doped porous carbon (N- PC) and single, double or multi-layer CNTs.
4. Give recommendations for application of the obtained materials as electrodes for supercapacitors and lithium-ion batteries.

## 2. Materials and Methods

The experimental work consists of the synthesis of multilayer carbon nanotubes (MWNTs). The synthesis was performed using two types of catalysts. The catalysts were prepared via combustion (catalyst 1) and deposition (catalyst 2). In the course of the study, the effect of the rate and ratio of CH_4_:H_2_ flows on the structure of carbon nanotubes (CNTs) was studied. Additionally, in the course of the work, a hybrid material based on porous carbon (PC) was obtained. The porous carbon was mixed with various nanotubes. Single-layer carbon nanotubes (SWCNTs), double layer carbon nanotubes (DLCNTs), and multilayer carbon nanotubes (MWCNTs) were used. The testing of the obtained hybrid materials was carried out when they were used as electrodes. The electrodes were used in Li-ion batteries and supercapacitors.

### 2.1. Materials and Reagents

The following reagents were used for the synthesis of Co polymolybdate catalyst: hydrazine sulfate N_2_H_4_-H_2_SO_4_ (HDA), ammonium heptamolybdate (NH)_46_ Mo O_724_-4HO_2_ (Cl), acetic acid CH_3_COOH (Cl), and cobalt acetate (CH_3_COO)_2_Co-4H_2_O (Cl). To prepare catalyst 1, cobalt polymolybdate was applied to magnesium oxide MgO (Cl). To synthesize catalyst 2, we used the following reagents: magnesium nitrate Mg(NO_3_)_2_∙6H_2_O (Cl) and citric acid C_6_H_8_O_7_. The following gases were used for the synthesis of MWNT: Ar (99.998 vol.%), CH_4_ (99.999 vol.%), and H_2_ (99.999 vol.%). HCl (HCl) was used to purify the MWNTs from the catalyst. Teflon and H_2_SO_4_ (1 M) were used to prepare electrodes for electrochemical studies in supercapacitors. OCSiAl SWCNTs, DUNTs, porous carbon (PC), and nitrogen-containing porous carbon (N-PC) synthesized at the National Chemical Laboratory (Pune, India) were used to prepare hybrid materials. Foam nickel, N-methylpyrrolidone, and fluoroplastic-2 were used to prepare electrodes for casting intercalation. Li, 1 M solution of LiPF6 in a mixture of ethylene carbonate and dimethyl carbonate (1:1) was used for casting intercalation.

### 2.2. Synthesis of CNT Using Precipitation and Combustion Catalysts

#### 2.2.1. Preparation of Catalysts by Precipitation and Combustion Methods

The synthesis of cobalt polymolybdate (Mo_12_O_28_(μ_2_-OH)_12_{Co(H_2_O)_3_}_4_) was performed via the method from [15]. The synthesis was carried out in several stages: (NH_4_)_6_Mo_7_O_24_∙4H_2_O (3.4 g) and Co(OOCCH_3_)_2_∙4H_2_O (11.3 g) were dissolved in 250 mL water and 50 mL acetic acid. Hydrazine sulfate (0.6 g) was added to the resulting solution while actively stirring. The resulting green solution was stirred for 10 min and heated in an Erlenmeyer flask for 3 days in an oil bath at 67 °C. The resulting red-orange crystals were washed with water after filtration [5].

Methodology for the application of polymolybdate is as follows: The Co polymolybdate was slowly stirred on a magnetic stirrer for 10 min until a stable suspension was formed. Then a carrier was added; magnesium oxide MgO was chosen as a carrier because it has a developed specific surface area and is easily removed with hydrochloric acid after catalytic chemical vapor deposition. The resulting mixture was stirred for 10 min until a stable homogeneous suspension was formed and then left at 60 °C until complete evaporation [5].

Catalyst 1 was obtained according to the method denoted in [5]. Magnesium nitrate Mg(NO_3_)_2_, cobalt polymolybdate, and ammonium heptamolybdate (NH_4_)_6_Mo_7_O_24_ were dissolved in citric acid. The resulting solution was placed in a muffle furnace heated to 540 °C. A catalyst powder (Catalyst 1) was obtained via “thermal shock”.

#### 2.2.2. Synthesis of Carbon Nanotubes by Catalytic Chemical Vapor Deposition

To synthesize carbon nanotubes (CNTs) by catalytic chemical vapor deposition (CCVD), Co polymolybdate deposited on MgO was decomposed in a muffle furnace at 700 °C for 11 min and then cooled to room temperature. The decomposition of Co polymolybdate leads to catalyst activation, in which catalytic MoO_3_ and CoMoO_4_ particles with an average size of five nm are formed [5].

**Figure 2:**
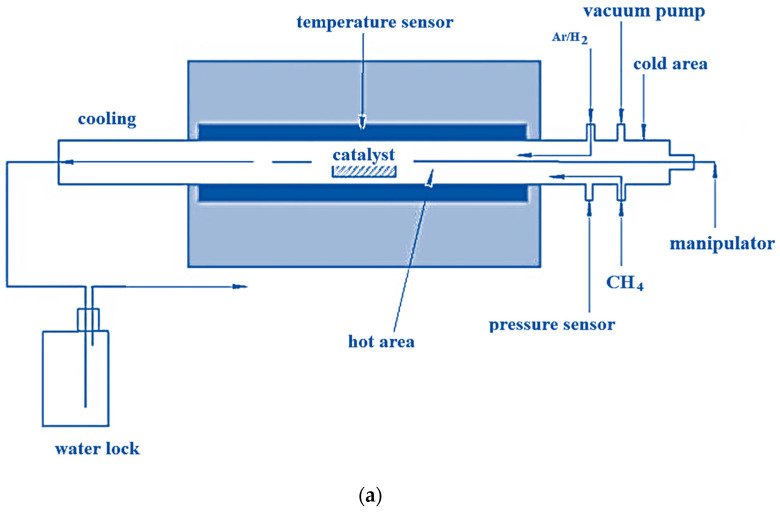

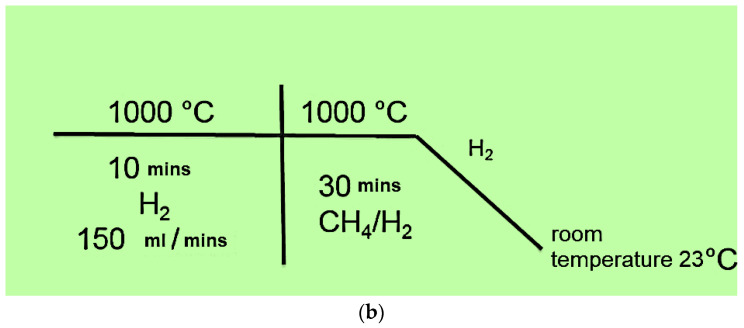
(**a**) Scheme of the CVD reactor; (**b**) Schematic of CCVD synthesis of CNT.

A tubular reactor was used for CNT synthesis, the scheme of which is shown in Figure 2a. The reactor casing is a stainless steel tube with a removable quartz tube 30 mm in diameter and 1 m long inserted inside. To one end of the reactor vessel is attached a feed block, which has outputs through which various substances (inert gas, carbon source, hydrogen, etc.) and a pressure sensor are injected. A movable manipulator is connected through the feed block, which allows the introduction of the catalyst into the center of the quartz tube at any time of the process. The reactor volume is evacuated through the feeder unit. A thermos regulator controls the temperature in the reactor. A schematic of CCVD synthesis of CNT is shown in Figure 2b.

A vacuum system is required to quickly evacuate air from the device and check the tightness of the CCVD reactor. The tube furnace provides heat in the central zone of the reactor in the range from room temperature to 1000 °C (Figure 2b). The gas flows were controlled using gas flow controllers ranging from 10 to 1500 mL/min. Carbon-containing compounds could be fed directly from a cylinder. The reactor outlet is connected to a liquid gate. The gaseous products resulting from CNT synthesis enter the exhaust ventilation system. The pH value at 25 °C is 4.

To synthesize CNTs, powders of catalyst 1 or catalyst 2 were evenly distributed on a ceramic boat. The boat was placed in the cold zone of the reactor. The reactor volume was evacuated, filled with hydrogen, and heated to 1000 °C. The synthesis took place at a constant temperature profile. Once the set temperature was reached, the ceramic boat was introduced into the hot zone of the reactor using a manipulator. To activate the catalyst-reduction to metallic particles, the reactor was purged with hydrogen at a rate of 150 mL/min for 10 min. CNT synthesis was carried out under constant hydrogen and methane flow rates for 30 min. At the final stage of synthesis, the reactor was purged with hydrogen for 10 min (150 mL/min). During the research work, a series of CNT syntheses with different ratios of CH_4_ and H_2_ flow rates were made.

During synthesis, a black outgrowth of carbon material formed on the surface of the ceramic boat. The CNT samples were washed from the catalyst particles and MgO with concentrated hydrochloric acid under stirring for 4–6 h. Then, the carbon material was filtered and repeatedly washed with distilled water, after which the CNT samples were dried in a desiccator for 6–12 h at 100 °C [16].

## 3. Experimental Section

The influence of the catalyst type on the structure of carbon nanotubes is documented here. In addition to the catalyst, the effect of CH_4_/H_2_ flux rates and concentration ratios was studied. The nanotubes under study were fabricated via CCVD. Raman spectroscopy and transmission electron microscopy were chosen as investigation methods [16,17].

**Figure 3:**
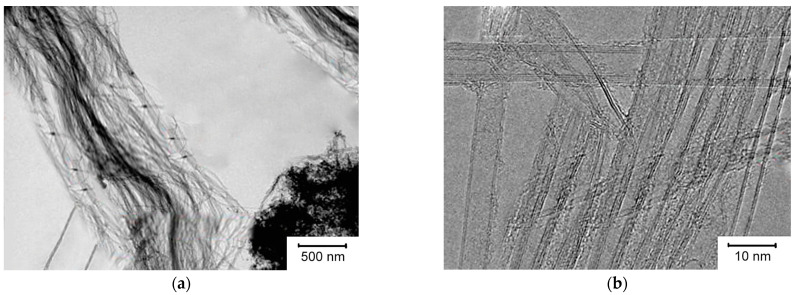
Low- and high-resolution TEM microphotographs of CNTs, respectively, synthesized using catalyst 1 at the flow rate of methane 200 mL/min; (**a**)—bilayer carbon nanotube fibers; (**b**)—bilayer carbon nanotube fibers vertically and multilayer carbon nanotubes horizontally.

Images of carbon materials obtained by transmission electron microscopy (TEM) are shown in Figure 3. Catalyst 2 was used to make these carbon materials. The carbon materials consist of balls and bundles of CNTs and do not contain amorphous carbon. A high-resolution TEM image shows that the sample (the flow rate when it was obtained was CH_4_ of 200 mL/min) contains bundles of bilayer carbon nanotubes (DNTs). For these nanotubes, the outer diameter is 2–5 nm. The agglomerates contained individual MWNTs. The MWNTs obtained at a flow rate of 200 mL/min CH_4_ had an average diameter of 5 nm. These MWNTs were the thinnest.

**Figure 4:**
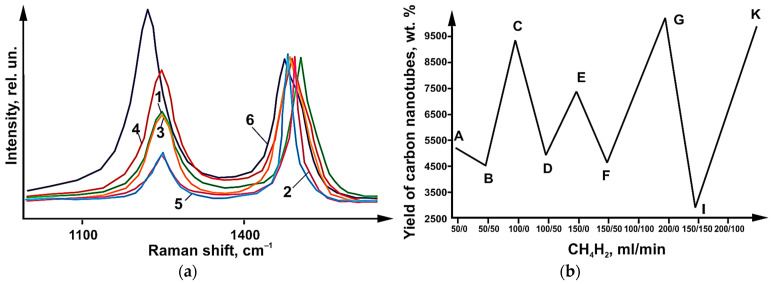
(**a**) CRT spectra of carbon nanotubes synthesized using catalyst 1 at total CH_4_/H_2_ flow rates 50 (1—50 CH_4_/50 H_2_), 100 (2—100 CH_4_; 3—100 CH_4_/50 H_2_), 150 (4—150 CH_4_), 200 (5—200 CH_4_) and 300 mL/min (6—300 CH_4_). (**b**) Effect of the ratio and flow rates of CH_4_/H_2_ gases on the CNT yield and I value S_D_/I_G_.

Raman light scattering (LBS) spectra of synthesized CNTs (Figure 4) revealed the presence of a peak. On these spectra, a G-type peak appeared at 1610 cm^−1^. This peak corresponds to tangential vibrations in the graphite grid of carbon atoms. A D-peak was also found. This peak shows the defectiveness of the structure. It was found at 1290 cm^−1^ value. The integral ratio I_D_/I_G_ intensities for G- peaks and D- peaks make it possible to estimate the degree of defectiveness of carbon materials. This ratio works especially well for sp2-hybridized materials.

Figure 4b shows data on the yields of carbon nanotubes. Likewise, Figure 3b shows the I_D_/I_G_ values. These data are plotted for different values of CH_4_/H_2_ flow rates. CH4 flow rates of 200 mL/min yield the lowest ID/IG values (0.5). CH_4_ flow rates of 300 mL/min yield the highest I_D_/I_G_ values (1.6). A decrease in the yield of carbon nanotubes was observed when hydrogen was injected. This also produced no change or decrease in I_D_/I_G_ values (Figure 4b). A steady state was reached at values of 200 mL/min for the flow rate (CH_4_). An increase in the flow rate up to 300 mL/min resulted in the appearance of thick and defective carbon nanotubes.

It can be concluded that an effective synthesis condition is a flow rate of 200 mL/min CH_4_, which provides a high yield of CNTs and low-defect structure. As shown by experimental work, it is possible to reduce the yield of carbon nanotubes by diluting methane with hydrogen. Dilution at a ratio of 1:2 and 1:1 give a reduction in the yield of tubes. At the same time, the structure of CNTs is improved in the presence of hydrogen. This was achieved at total CH_4_:H_2_ flow rates 300 mL/min and 150 mL/min. At other flow rates, the addition of H_2_ had no effect on the defectiveness of the CNT structure. Hydrogen in this case served two functions. One was the dilution of methane. Its second function was to stabilize methane during its decomposition.

**Figure 5:**
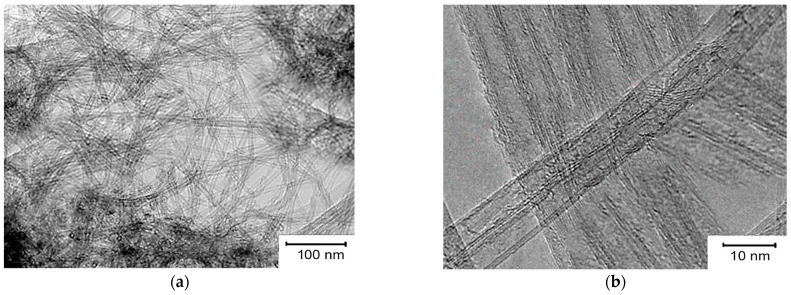
Low- and high-resolution PET images of DUNTs, TUNTs, and MWNTs, respectively, synthesized using catalyst 1 CH_4_ at flow rates of 200 mL/min; (**a**)—multilayer carbon nanotube fibers; (**b**)—bundles of bilayer carbon nanotubes.

The following are the results of a study of the structure of CNTs synthesized on a catalyst obtained via combustion. Figure 5 shows low-resolution PET images of highly dispersed thin CNTs obtained using catalyst 1 at a flow rate of 200 mL/min. The high-resolution PEM images show the presence of CNTs, triple-layer CNTs (TUNTs), and MWNTs in the sample. The average outer diameter of these CNTs was 3, 5, and 7 nm, respectively. Also, MWNTs with a large outer diameter up to 14 nm were observed in the sample.

**Figure 6:**
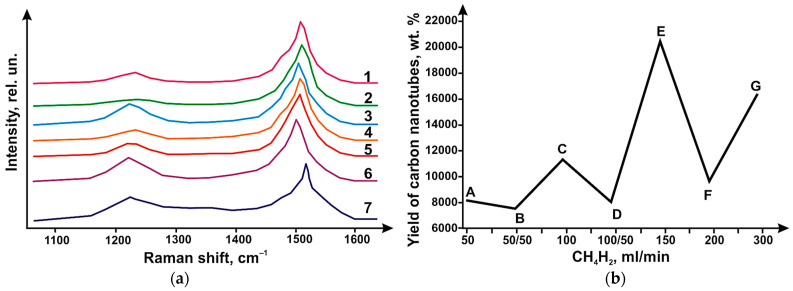
(**a**)-CRT spectra of synthesized carbon nanotubes (using catalyst 1) at different flow rates of CH_4_/H_2_. The graph shows the following flow rates: 50 (1—50 CH_4_; 2—50 CH_4_/50 H_2_), 100 (3—100 CH_4_; 4—100 CH_4_/50 H_2_), 150 (5—150 CH_4_), 200 (6—200 CH_4_) and 300 mL/min (7—300 CH_4_); (**b**)-CNT yield depending on the ratio and flow rates of CH_4_/H_2_.

The result of this research work was the determination of the I_D_/I_G_ ratio of the CRS spectra of carbon nanotubes. These ratios varied in the range of 0.2–0.8, which is clearly seen in Figure 6. These values are lower than the values (Figure 3b) obtained with catalyst 1. The CNT yields are shown in Figure 6b. The yield is shown as a function of the CH_4_/H_2_ gas flow rates at different ratios. At hydrogen saturation, the CNT yield decreased, as in the case of catalyst 1. The highest CNT yield was obtained at a flow rate of 150 mL/min (for CH_4_ flow). At a flow rate of 50:50 mL/min for the methane–hydrogen flow rate, the lowest CNT yield was obtained. For the highest CNT yield, the I_D_/I_G_ ratio was 0.5. and 0.2 for the case where the yield was minimal. The highest I_D_/I_G_ ratio was achieved when the methane flow rate increased to 300 mL/min. In this case, there is a deviation from the steady state. As a result, the number of bilayer nanotubes formed decreases. The share of multilayer tubes of large diameter grows. A similar situation was observed with catalyst 1. In Figure 6a, the presence of RBM (radial breathing mode) peaks is observed in all samples, which indicates the formation of both single- and double-layer carbon nanotubes regardless of the flow rates and the CH_4_/H_2_ ratio. Pure and hydrogen-free methane yielded nanotubes with a small variation in diameter. This is characteristic of methane flow at low velocities (Figure 6). Figure 6 also shows that the decrease in carbon nanotube yield comes at CB (100 mL/min), ED (150 mL/min), and GF (300 mL/min) segments. This happens when hydrogen dilutes methane.

It follows from the above that CH_4_ has sufficient reducing ability to form metal particles. The size of these particles is sufficient for the growth of DNT [18,19]. The addition of hydrogen to methane increases the reducing ability. As a result, catalytically active particles are formed. The sizes of these particles differ from the previous ones [20,21]. As a result, nanotubes of various sizes are formed.

During the study of the electrochemical characteristics of CNTs, the obtained CNTs were tested as electrodes for supercapacitors. The technology of carbon-based electrode creation consists of the mixing and homogenization of carbon materials with a binder (Teflon F-4D) in ethyl alcohol. The resulting electrode is a film 200–300 µm thick with an area of 1 × 1 cm^2^. The cell is a film of carbon material sandwiched between platinum current collectors separated by a polypropylene fiber membrane. The Ag/AgCl chlorosilver electrode acted as a reference electrode. The electrolyte used was 1 M H_2_ SO_4_.

The results of the electrochemical experiment were obtained as cyclic volt-amperograms (CVA) recorded in a potential window from 0 to 1 V at a stepwise change in the potential sweep rate of 2–1000 mV/s. The calculation of the specific heat capacity of the electrodes was carried out using the formula C = S/(Vs × m). In this formula, S is the area under the positive curve, Vs is the sweep speed, and m is the total mass for the carbon material used.

**Figure 7:**
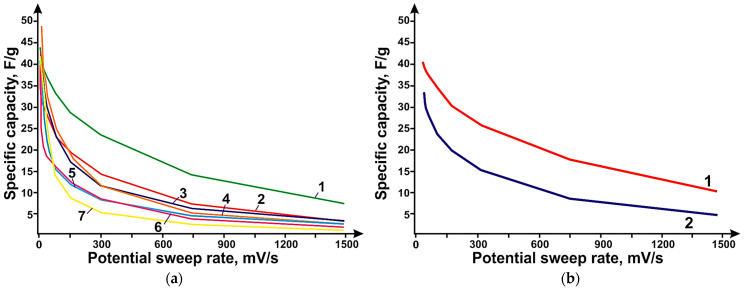
Dependence of specific capacity: (**a**) CNTs synthesized using catalyst 1 at total flow rates of methane/hydrogen mixture. The following flow rates are plotted: 50 (7—50 CH_4_; 4—50 CH_4_/50 H_2_), 100 (5—100 CH_4_; 3—100 CH_4_/50 H_2_), 150 (1—150 CH_4_), 200 (2—200 CH_4_) and 300 mL/min (6—300 CH_4_); (**b**) CNTs synthesized using catalyst 1 and 2 at CH flow rate_4_ 200 mL/min; curve 1 is catalyst 2; curve 2 is catalyst 1.

The studies showed that the sample obtained at a methane flow rate value of 150 mL/min on catalyst 1 had the highest specific capacity over the entire range of sweep rates. Carbon nanotubes obtained using catalyst 1 showed higher values of specific capacitance over the whole range of sweep rates compared to nanotubes synthesized using catalyst 1, which may be due to the higher specific surface area, which are 379 and 285 m^2^/g, respectively. Cyclic volt-amperogram curves show peaks at ~350 mV. These peaks appear on the charge curve at ~550 mV. On the discharge curve, they appear at ~240 mV. These values are associated with reduction of molybdenum (Mo^+6^) to its intermediate oxides (MoOx) and correspond to redox processes. For the stopping molybdenum oxides, x is between 2.4 and 3.2. Figure 7 shows the dependence of specific capacity, where CNTs synthesized using catalyst 1 at total flow rates of methane/hydrogen mixture; (b) CNTs synthesized using catalyst 1 and 2 at flow rates of CH_4_ 200 mL/min.

The hybrid materials were created as follows. All samples do not contain any amorphous carbon impurities. SWCNTs are mainly bundles of single-layer CNTs with a diameter of 10–100 nm and an admixture of individual SWCNTs with an outer diameter of 1.7–1.9 nm. Bundles of SWCNTs with an outer diameter of 2.1 to 3.8 nm were obtained. In the MWNT sample synthesized earlier, the outer diameter of the CNT is 5 nm. The PC was obtained by heating MgO in a CH_4_ stream under dynamic temperature profile conditions to 900 °C at a heating rate of 15 °C/min. After reaching the target temperature, the synthesis was continued for 6 min, then the reactor was cooled in the CH stream. After reaching 120 °C, the system was purged with N_2_ for one hour. Nitrogen-doped porous carbon (N-PC) MgO was obtained by heating MgO in the N_2_/CH_3_ CN flow at a heating rate of 15 °C/min to a temperature of 900 °C. Once the target temperature was reached, the synthesis was continued in the CH_4_/CH_3_ CN flow for 6 min. The reactor was then uncontrollably cooled in the CH_4_/CH_3_ CN flow to 120 °C, then purged with N_2_ for one hour. N-PC (CaO) was obtained by heating CaO in a stream of N_2_/CH_3_ CN at a rate of 15 °C/min to a temperature of 900 °C. The synthesis was continued in the CH stream_4_ at 900 °C for 6 min. Then the reactor was uncontrollably cooled in the CH_4_/CH_3_ CN flow to 120 °C, after which it was purged with N_2_ for one hour.

In general, PC should have a developed specific surface area and a high content of micro- and mesopores, which allows the electrolyte to penetrate the material, thereby improving electrolyte diffusion. The use of N-PC, as compared to PC, can increase the efficiency of the electrodes due to the presence of a nitrogen heteroatom in the structure, thus increasing the conductivity of the material as well as providing additional sorption sites. CNTs are used as a “conducting” link between individual particles of porous carbon.

The method of preparation of materials for the creation of electrodes was perfected on CNT and N-PC (CaO) samples. The samples were dispersed in water via ultrasonic treatment in an ultrasonic bath (power 100 W) for one hour. Subsequently, the aqueous medium was dispersed using the following procedures:

- Aerosol spraying of dispersions. Carbon material dispersions were sprayed directly onto the platinum electrode, which is used in electrochemistry as a current collector, using an airbrush “JAS”.
- Thermal treatment. The carbon material dispersions were thermally dried at 80 °C for 12 h.
- Lyophilic drying. Carbon material dispersions were frozen at liquid nitrogen temperature and subjected to lyophilic drying, which promotes additional dispersion of samples due to the transition of water from solid to gaseous state.

As a result of this research, the electrochemical characteristics of the hybrid materials were determined. In order to evaluate the efficiency of the hybrid materials, the individual components were tested as electrodes for supercapacitors.

**Figure 8:**
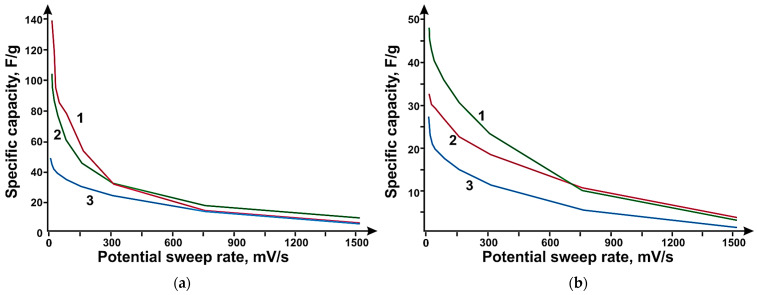
Dependence of specific capacity of electrodes based on: (**a**)—PC (Curve 3), N-PC (CaO) (Curve 2), N-PC (MgO) (Curve 1); (**b**)—on CNT (Curve 3), DLCNTs (Curve 1) and MWNT (Curve 2) on potential sweep speed.

The most effective method of getting rid of water, both in the case of SWCNTs and N-PC (CaO), was lyophilic drying, which contributes to additional dispersion of samples due to the transition of water from solid to gaseous state. Electrodes based on SWCNTs and N-PC (CaO) obtained by lyophilic drying have a specific capacity of 29 F/g and N-PC (CaO) of 111 F/g at a sweep rate of 2 mV/s. In Figure 8a, the PC, N-PC (CaO), and N-PC (MgO) samples show capacities of 51, 111, and 148 F/g at 2 mV/s, respectively, after lyophilic drying. The introduction of a nitrogen heteroatom into the structure of porous carbon yields a significant increase in the efficiency of the electrodes due to an increase in the conductivity of the material. N-PC (MgO) is more efficient compared to N-PC (CaO), presumably because of the large amount of incorporated nitrogen in the N-PC structure, which is possibly due to the synthesis features as well as the size of N-PC (MgO) particles. In Figure 8b, the electrodes based on SWCNTs, MWNTs, and DLCNTs s show capacities of 29, 33, and 48 F/g at 2 mV/s, respectively.

The hybrid material was created by mechanically mixing the components in different proportions, dispersion in water, and further lyophilic drying. By adding more than 5 wt% SWCNT, the specific capacitance of the hybrid material decreases from 18 to 80% at 2 mV/c. The addition of 1 wt% SWCNTs does not result in any change in specific capacity compared to pure N-PC (CaO). Thus, the addition of 5 wt% of SWCNTs to N-PC (CaO) is the most optimal and increases the capacity of the hybrid material compared to the capacities of the individual components. SWCNTs also act as a “conducting” link between the individual particles of porous carbon, which increases the conductivity of the hybrid material and, at the same time, keeps the structure of porous carbon unchanged and the pores available for electrolyte ions in contrast to samples with a percentage of SWCNTs above 5 wt%, which show a reduction in the specific electrode capacity over the entire range compared to pure N-PC (CaO). The increase in the capacity to 265 F/g compared to the original graphene (180 F/g) was carried out with the addition of 16 wt% SWCNTs. With the increase of the MWNT content in the hybrid material, the specific capacity of the hybrid material electrodes decreased by 10% compared to graphene.

Based on the experiments performed, the 94:6 wt% component ratio in the hybrid material was selected for further investigation. Hybrid materials based on PC or N-PC (MgO), N-PC (CaO) combined with SWCNTs, DLCNTs, and MWNTs in a 94:6 wt% ratio were tested as electrodes for supercapacitors

**Figure 9:**
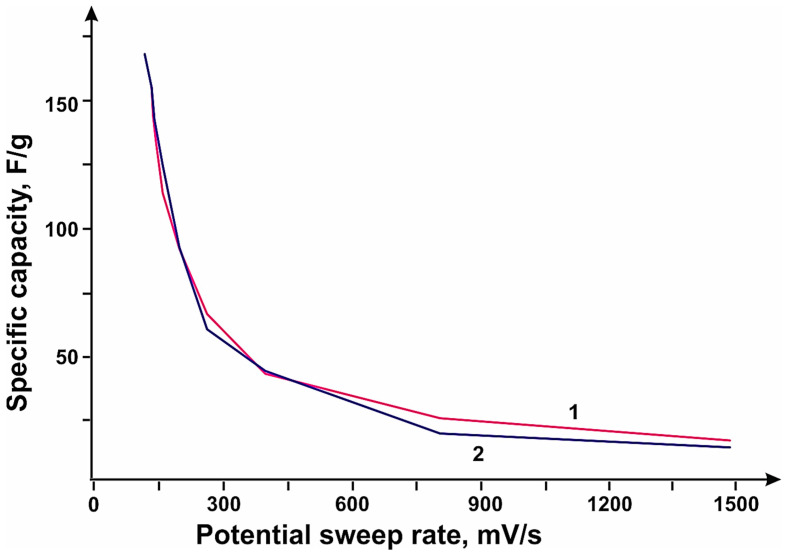
Dependence of the specific capacity on the reversal rate of the N-PC potential (Curve 1) and N-PC (MgO):SWCNT 95:5 wt% (Curve 2).

Figure 9 shows cyclic volt-amperogram curves at a potential sweep rate of 5 mV/s. At low sweep rates, we observe peaks on the charge curve at 450 mV/s and on the discharge curve at 425 mV/s, which correspond to oxygen-containing functional groups on the surface of the hybrid material. Since SWCNTs are conductive additives, one can observe that the area of the CVA curve of the hybrid material is larger than that of the original N-PC (MgO).

To describe the electrochemical processes of N-PC (MgO)/OUNT, DLCNT, and MWNT hybrid materials, the porous structure of the hybrid materials was analyzed via nitrogen adsorption using a “Sorbi MS” instrument. Before the measurement, the carbon material was annealed in an inert atmosphere using “Sorbi-MS” equipment at 160 °C for 1.5 h.

**Figure 10:**
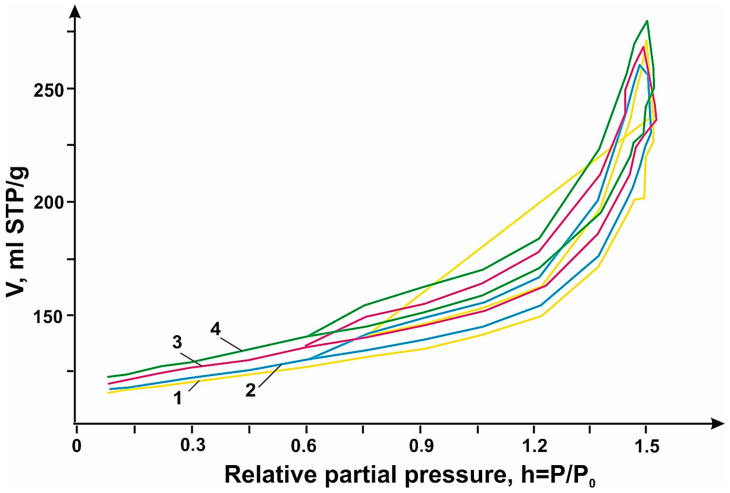
Nitrogen adsorption-desorption isotherm of N-PC (MgO) (Curve 4), N-PC (MgO):DNT (Curve 3), N-PC (CaO):MWNT (Curve 2), N-PC (CaO):SWCNT (Curve 1) samples.

The nitrogen adsorption–desorption isotherm (Figure 10) was measured between 0.06 and 0.99 relative partial pressure. The specific surface area was measured via the Brunauer–Emmett–Teller (BET) method, which for the materials N-PC (MgO), N-PC (MgO): DLCNT, N-PC (MgO):MWNT, and N-PC (MgO):SWCNT are 253, 230, 196, and 182 m^2^/g, respectively. The electrochemical capacity of carbon materials is determined by the effective surface area available for electrolyte ions. Hybrid materials are the most efficient compared to the original N-PC (MgO) in electrochemistry, despite the smaller surface area. This is because the electrochemical surface area is larger than the estimated specific surface area by gas adsorption because the voltage-controlled mechanism can facilitate the access of electrolyte ions into the spaces between the carbon layers. Consequently, when CNTs were added, there was a decrease in the surface area of the hybrid material relative to the original N-PC, but the electrochemical area of the hybrid material increased, thereby improving the electrochemical performance.

In order to determine the properties of hybrid materials in a lithium-ion battery, the following scheme was used. N-PC (CaO) with SWCNTs, SWCNTs, and MWNTs and N-PC (MgO) with SWCNTs, DLCNTs, and MWNTs were chosen to test hybrid materials during Li-ion intercalation.

**Figure 11:**
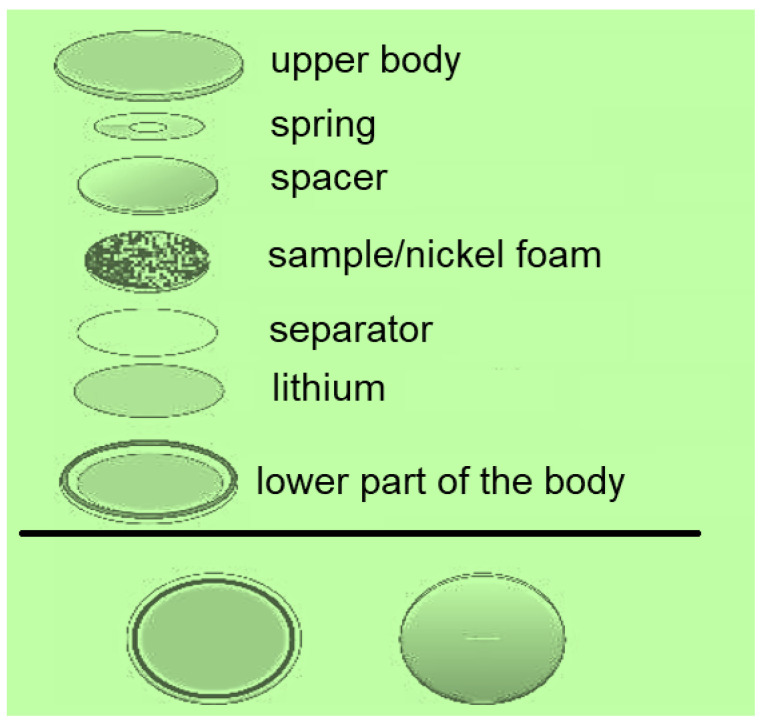
Schematic of the cell assembly for testing.

The technology of making electrodes for measuring the charge–discharge processes in lithium-ion batteries consisted of the application of carbon material to the foam-nickel acting as a current collector. For this purpose, the carbon material was homogenized with the binder polyvinyl difluoride (PVDF-2) in N-methylpyrrolidone. Afterward, the resulting mixture was smeared on the foam nickel and dried at 60 °C for 12 h. During the last stage, the electrodes were compressed and two-electrode metal cells (CR 2025 form factor) were assembled. Testing of obtained electrodes in lithium-ion battery was carried out using “Neware BTS-5000” charging–discharging stations in galvanostatic mode from 0.01 to 2.50 V with respect to Li/Li^+^ at different current densities by the following procedure: 10 cycles at current densities of 100, 250, 500, 1000, 100 mA each. The layout of the cell assembly for testing is shown in Figure 11.

**Figure 12:**
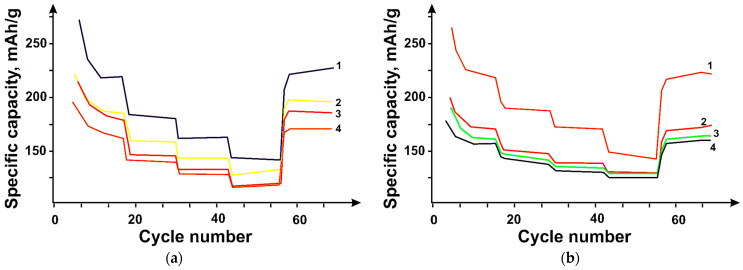
Changes in the specific capacity of carbon materials when the current densities change 100 mA/g, 250 mA/g, 500 mA/g, 1000 mA/g, 100 mA/g for hybrid materials (**a**) based on N-PC (CaO) (Curve 3), N-PC (CaO)/OUNT (Curve 4), N-PC (CaO)/DUNT (Curve 1), and N-PC (CaO)/MUNT (Curve 2) with a component ratio of 95:5 wt%, (**b**) based on N-PC (MgO) (Curve 4), N-PC (MgO)/OUNT (Curve 2), N-PC (MgO)/DUNT (Curve 1), and N-PC (MgO)/MUNT (Curve 3) with a 95:5 wt.

The cyclability of the hybrid material samples at different current densities is shown in Figure 12. The tested samples showed good long-term cyclability with almost 100% efficiency. The N-PC (CaO)/DNT 95:5 wt% and N-PC (MgO)/DNT 95:5 wt% samples showed the highest efficiency in Li-ion intercalation, 330 and 533 mAh/g after 10 cycles at a current density of 100 mA/g at the end of the experiment.

The N-PC (MgO)/DNT sample also has the highest pore distribution relative to its total volume, which is 0.491 cm^3^/g due to the structure formed during the hybrid material production, which contributes to improved lithium diffusion due to additional pores available for the ions.

As can be seen, the pore size of the hybrid materials varies from 4 to 86 nm (Figure 9), corresponding to a mesoporous structure. The high performance of the electrodes in injection-molding intercalation is provided by the wide pore size distribution, which facilitates diffusion and ion exchange during charging/discharging. Meanwhile, the N-PC (MgO)-based sample showed the highest efficiency among all the samples, which is probably due to the effect of incorporated nitrogen, which increases the number of sorption sites for Li. It should be noted that among CNTs, DUNTs were the most efficient, which is due to the presence of additional pores in DUNT bundles, which provide additional pores in the hybrid material suitable for reversible Li incorporation [22,23].

The addition of 1 wt% SWCNT does not lead to any changes in specific capacity compared to pure N-PC (CaO). Thus, the addition of 5 wt% of SWCNTs to N-PC (CaO) is the most optimal and increases the capacity of the hybrid material compared to the capacities of individual components. Additionally, SWCNTs act as a “conducting” link between individual particles of porous carbon, which increase the conductivity of the hybrid material and, at the same time, allow the structure of porous carbon to remain unchanged and pores to remain accessible to electrolyte ions in contrast to samples with the percentage of SWCNTs above 5 wt%, in which there is a decrease in specific electrode capacitance in the whole range in comparison with pure N-PC (CaO).

With an eye to the future development of batteries and supercapacitors, it should be noted that the creation of hybrid materials is an effective way to improve the electrochemical characteristics of lithium-ion batteries and supercapacitors, such as high electric capacity, battery current output, and reduction of battery degradation during operation. Obtaining hybrid materials by mechanical mixing makes it possible to control the composition of the material, thereby setting the optimal characteristics of this material [22,23,24].

## 4. Main Results and Conclusions

- This work investigated the effect of CCVD synthesis parameters, such as flow rate, methane-to-hydrogen ratio, and type of catalytic system on the structure of multilayer carbon nanotubes (MWNTs). MWNTs were synthesized and two types of Mo_12_O_28_ (μ_2_-OH)_12_{Co(H_2_O)_3_}_4_-based catalysts on MgO carriers were prepared by deposition (catalyst 1) and combustion (catalyst 2) methods to develop a technique for obtaining a hybrid carbon material based on porous carbon (H1U) or nitrogen-doped porous carbon (N- PC) and single-, double- or multi-layer CNTs. Recommendations are given for the application of these materials as electrodes for supercapacitors and lithium-ion batteries.
- Cobalt polymolybdate Mo_12_O_28_ (μ_2_-OH)_12_{Co(H_2_O)_3_}_4_ was used as an initial substance for the production of catalysts by combustion and impregnation synthesis methods for the carbon nanotubes. It was shown that polymolybdate distributed on the surface of the MgO carrier allows predominantly multilayer CNTs (MWNTs) to be obtained. The catalyst obtained by combustion-MWNTs with an admixture of single-layer CNTs (SWCNTs) and double-layer CNTs (DSNTs) can produce methane-hydrogen. Methane-hydrogen is obtained regardless of the ratio of fluxes from the velocities.
- The influence of methane (50–300 mL/min) and hydrogen (0–150 mL/min) flow ratios and their velocities on the structure of carbon nanotubes was determined. Carbon nanotubes were obtained using different catalysts. It was shown that the addition of hydrogen to methane reduces the density of defects in carbon nanotubes.
- The effect of the mass ratio of the components (porous carbon and carbon nanotubes) of hybrid materials on the electrochemical properties of electrodes based thereon has been shown. The introduction of 5 wt% CNT addition to porous carbon leads to improvement of electrochemical characteristics of electrodes based on them: by 6% for PC/OUNT compared to PC at 2 mV/c, by 10% for N-PC (CaO)/OUNT compared to N-PC (CaO) at 2 mV/c and 143% at 1000 mV/c, by 10% for N-PC (MgO)/OUNT compared to N-PC (MgO) at 2 mV/c.
- The influence of the hybrid material composition (porous carbon and carbon nanotubes) on the efficiency of lithium-ion batteries has been shown. The introduction of 5 wt% DST into the porous carbon leads to an improvement in the specific capacity of Li-ion batteries: by 141% for N-PC (MgO)/DNT compared to N-PC (MgO) and by 32 % for N-PC (CaO)/DNT compared to N-PC (CaO) at a current density of 100 mA/g.

## Figures and Tables

**Table 1:** The main characteristics of batteries.

Specification	Rechargeable Batteries			
	Li–Ion	Lithium– Polymer Lipol	Pb Acid	Nickel– Metal Hydr Idnys Ni-MH
Weight (kg)	2.15	2	10	5.5
Specific energy (Wh/kg)	280–400	280–450	30–60	60–72
Threshold state of charge	80%	40%	50%	50%
Working temperature	−20 °C …+40 °C	−20 °C …+40 °C	−40 °C …+40 °C	−60 °C …+55 °C
Efficiency	100% at 20	-	100% at 20	-
	opening hours;		opening hours;	
	99% at 4 h		80% at 4 h	
	work;		work;	
	92% at 1 h		60% at 1 h	
	work		work	
Voltage (V)	3.2–4.2	3.2–4.2	2.11–2.17	1.2–1.25
Environmental friendliness	Yes	Yes	No	Yes

## Data Availability

The data presented in this study are available from the corresponding authors upon reasonable request.
